# Additive Effect of Sarcopenia and Anemia on the 10-Year Risk of Cardiovascular Disease in Patients with Type 2 Diabetes

**DOI:** 10.1155/2022/2202511

**Published:** 2022-01-24

**Authors:** Feihui Zeng, Lingning Huang, Yongze Zhang, Xinyu Hong, Suiyan Weng, Ximei Shen, Fengying Zhao, Sunjie Yan

**Affiliations:** ^1^Department of Endocrinology, The First Affiliated Hospital of Fujian Medical University, 20 Cha Zhong Road, Fuzhou, Fujian 350005, China; ^2^Clinical Research Center for Metabolic Diseases of Fujian Province, The First Affiliated Hospital of Fujian Medical University, 20 Cha Zhong Road, Fuzhou, Fujian 350005, China; ^3^Diabetes Research Institute of Fujian Province, The First Affiliated Hospital, Fujian Medical University, 20 Cha Zhong Road, Fuzhou, Fujian 350005, China; ^4^Metabolic Diseases Research Institute, The First Affiliated Hospital of Fujian Medical University, 20 Cha Zhong Road, Fuzhou, Fujian 350005, China

## Abstract

**Objective:**

To investigate the association between sarcopenia and anemia and the 10-year cardiovascular disease risk in diabetic patients.

**Methods:**

A cross-sectional study was conducted involving 4673 hospitalized patients (2271 men and 2402 women) with type 2 diabetes mellitus, with an average age of 60.66 ± 11.93 years, of whom 542 were followed up for a median follow-up period of 24 months. All participants underwent body composition measurements, and they were grouped by sex and presence of sarcopenia using the Framingham risk model to assess their 10-year cardiovascular risk. According to the changes in the cardiovascular risk during follow-up, the patients were divided into four groups: low-low, low-high, high-low, and high-high.

**Results:**

The prevalence of anemia was higher in the sarcopenia group than in the nonsarcopenia group (11.5% vs. 24.1% for men, *P* < 0.001; 13.9% vs. 19.7% for women, *P* < 0.05), and the difference remained significant after adjusting for confounders. Patients with sarcopenia and without anemia had a 46.2% increased risk of high 10-year cardiovascular disease (CVD) (odds ratio (OR) = 1.462, 95% confidence interval (CI) 1.085–1.972, *P* = 0.013), and the risk was twofold higher in patients with sarcopenia and anemia than in those without (OR = 3.283, 95% CI 2.038–5.289, *P* < 0.001). In follow-up studies, sarcopenia was associated with an increased risk of CVD at 10 years, and a reduction in appendicular skeletal muscle mass index independently predicted the increased risk of CVD.

**Conclusion:**

Sarcopenia is associated with an increased risk of anemia, and the presence of both has an additive effect on the 10-year CVD risk in patients with type 2 diabetes. Loss of muscle mass can independently predict an increased CVD risk in diabetic patients.

## 1. Introduction

Sarcopenia is a syndrome characterized by the continuous loss of skeletal muscle mass, strength, and function [1], which is common in diabetic patients [2, 3] and has recently been identified as a chronic complication of diabetes [4]. Sarcopenia or low skeletal muscle mass has been shown to be significantly associated with a decreased renal function, sleep disorders, peripheral neuropathy, and an increased risk of all-cause death in diabetic patients [5–8]. Typically, the risk of cardiovascular disease (CVD) in diabetic patients is usually considerably increased, and sarcopenia is also associated with the risk of CVD [9]. However, to date, no studies have investigated the association between sarcopenia and the risk of CVD in diabetic patients.

Anemia, which can lead to several clinical diseases and adverse outcomes, affects almost a third of the world's population [10]. Anemia occurs in nearly 20% of diabetic patients and is associated with complications and poor prognosis. Previous studies have shown that anemia is an important cause of the progression of diabetic microvascular and macrovascular complications [11, 12], greatly affecting the quality of life of patients and increasing medical expenses. Meanwhile, anemia has been confirmed as an independent risk factor for cardiovascular events in patients with diabetic nephropathy [13].

Both sarcopenia and anemia are associated with malnutrition and have been identified as risk factors for cardiovascular events in several studies [14, 15]. With respect to sarcopenia, previous studies have emphasized its association with falls, weakness, and nutrition [16–18]. However, few studies have investigated the association between sarcopenia and anemia, particularly in relation to their impact on the risk of cardiovascular events. Good control over multiple risk factors for type 2 diabetes can reduce the risk of CVD [19]; therefore, it is necessary to explore the association between sarcopenia, anemia, and the 10-year risk of CVD in patients with type 2 diabetes. The purpose of this study was to investigate the following: (1) Are sarcopenia and anemia associated with CVD risk? (2) Will a reduction in the appendicular skeletal muscle mass index (ASMI) and hemoglobin (HGB) concentration increase the risk of CVD?

## 2. Materials and Methods

### 2.1. Study Population

**Figure 1:**
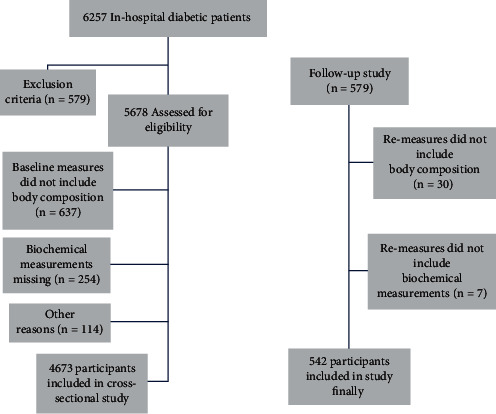
Flow diagram.

Participants were recruited from among inpatient populations from March 2007 to August 2019 by simple random sampling. The sample consisted of adults who had volunteered. Initially, 5,678 participants were assessed for eligibility and included in the survey. Only 579 participants participated in the follow-up study (Figure 1), because some of them were transferred back to local hospitals for treatment after discharge and were not followed up in our hospital. Some patients were not included in the analysis due to a lack of data on body composition, biochemical indicators, or other reasons. The sample size for the final study was thus 4,673 in the cross-sectional study and 542 in the follow-up study. The mean age of the 4,673 hospitalized patients with type 2 diabetes (2,271 men and 2,402 women) was 60.66 ± 11.93 years, and that of the 542 follow-up patients (282 men and 260 women) was 61.62 ± 10.33 years, with a median follow-up time of 24 months. The exclusion criteria included any of the following: type 1 diabetes, gestational diabetes, diabetic ketoacidosis, hyperosmotic nonketotic coma, heart disease, thyroid disease, chronic liver disease, severe renal insufficiency, active malignancy, long-term use of glucocorticoids, or pregnancy. Since the 24-month follow-up period was too short to assess the incidence of CVD, patients with a previous or current CVD were also excluded from the study. The study was approved by the ethics committee of the First Affiliated Hospital of Fujian Medical University, and written informed consent was obtained from the patients: MRCTA, ECFAH of FMU [2017]131.

### 2.2. Diagnostic Criteria

The diagnosis of type 2 diabetes was based on criteria established by the World Health Organization (WHO) in 1999 [20]. The body composition was determined by dual-energy X-ray absorptiometry (DEXA, Lunar Prodigy scanner, GE Lunar Corporation, Madison, WI, USA). Muscle mass was determined using ASMI, i.e., ASM (kg)/height (Ht) (m^2^). The diagnostic criteria for sarcopenia were based on the diagnostic strategies published by the International Working Group on Sarcopenia. The ASMI thresholds for men and women were set at 7.23 kg/m^2^ and 5.67 kg/m^2^, respectively [21]. Anemia was defined according to the parameters proposed by the WHO; therefore, the presence of anemia was indicated by HGB levels below 130 g/L in men and below 120 g/L in women [22]. The Framingham Risk Score (FRS) was used for assessing the 10-year CVD risk, based on the Framingham study by the National Heart, Lung and Blood Institute in Bethesda, Maryland, USA [23]. According to the Framingham algorithm summarized by D'Agostino et al., FRS was calculated using the scores corresponding to sex, age, total cholesterol (TC), high-density lipoprotein cholesterol (HDL-C), systolic blood pressure, smoking status, and presence or absence of diabetes [24], resulting in an FRS of ≥15 for men and ≥23 for women. The 10-year CVD risk ≥ 20% was considered to be high risk while that <20% as low risk. In the longitudinal study, the follow-up population was divided into low-low, low-high, high-low, and high-high groups according to the changes in the 10-year CVD risk from low to low, low to high, high to low, and high to high, respectively. According to the changes in ASMI, the variations in muscle mass were defined as ASMI decreased (ASMI variational rate < 3%), ASMI stabilized (3% ≤ ASMI variational rate ≤ 3%), and ASMI increased (ASMI variational rate > 3%) [25]. The variations in HGB were defined as HGB decreased (HGB variational rate < 7%), HGB stabilized (7% ≤ HGB variational rate ≤ 7%), and HGB increased (HGB variational rate > 7%) [26].

### 2.3. Measurements

In the morning, after 8 hours of fasting overnight, venous blood samples were collected from all participants for routine hematological and biochemical measurements: red blood cell count (RBC), HGB, mean corpuscular volume, mean corpuscular hemoglobin, mean corpuscular hemoglobin concentration, red cell distribution width, hematocrit (HCT), serum TC, triglyceride (TG), HDL-C, low-density lipoprotein cholesterol, albumin (ALB), glucose, creatinine (SCr), and blood urea nitrogen. The estimated glomerular filtration rate was estimated according to the Chronic Kidney Disease Epidemiology Collaboration (CKD-EPI) formula [27]. Glycosylated hemoglobin (HbA1c) levels were determined by high-performance liquid chromatography (VARIANT™ II, Bio-Rad, Hercules, CA, USA). The ratio of urinary albumin to urinary creatinine (UACR) (mg/g) was calculated by dividing the urinary microalbumin content with the urinary SCr content, and the results were classified as UACR < 30 mg/g and UACR ≥ 30 mg/g.

### 2.4. Statistical Analyses

The statistical data are expressed as mean ± standard deviation, median (P25–P75), or age. Chi-square tests for categorical variables and *t*-tests for continuous variables were used to compare the differences among the study groups. Multiple linear regression and binary logistic regression analyses were conducted to determine the association of sarcopenia with anemia, the 10-year CVD risk, ASMI, and hematological markers. *P* < 0.05 was considered statistically significant. All statistical analyses were conducted using the Statistical Package for the Social Sciences statistical software version 18.0 (Chicago, IL, USA).

## 3. Results

### 3.1. Baseline Characteristics of the Participants

A total of 4,673 hospitalized patients with type 2 diabetes were enrolled in this study, with an age range of 20–91 years (average, 60.66 ± 11.93 years) and average duration of diabetes of 8.13 ± 6.83 years; 2,271 men (average age, 59.38 ± 12.75 years) and 2,402 women (average age, 61.87 ± 10.97 years).  Table 1 presents the baseline characteristics of the participants, stratified by the diagnosis of sarcopenia. The sarcopenia group showed significantly higher age and HDL-C levels than the nonsarcopenia group, and the UACR was ≥30 mg/g in the sarcopenia group; the body mass index (BMI), diastolic blood pressure (DBP), TG, ALB, and calcium channel antagonist ratio were lower in the nonsarcopenia group than in the sarcopenia group (*P* < 0.05).

### 3.2. Association between Sarcopenia and Anemia

As shown in Table 1, anemia was more prevalent in the sarcopenia group (men, 24.1%; women, 19.7%) than in the nonsarcopenia group (men, 11.5%; women, 13.9%). In the multifactor logistics regression analysis, after considering the confounding factors such as age, duration of diabetes, diabetic complications, BMI, blood pressure, TC, TG, ALB, and SCr, smoking status, usage of hypoglycemic agents and hypotensors, and proportion of men and women, the sarcopenia group showed a higher risk of anemia than the non-sarcopenia group (men, odds ratio (OR) = 2.128, 95% confidence interval (CI) 1.501–3.018, *P* < 0.001; women, OR = 1.586, 95% CI 1.108–2.271, *P* = 0.012).

### 3.3. Association between ASMI and Hematological Indicators

**Figure 2:**
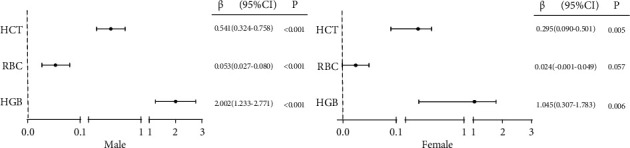
Forest plot of the multiple linear regression analysis of ASMI and HCT, RBC, and HGB. Abbreviations: ASMI: appendicular skeletal muscle mass index; HCT: hematocrit; RBC: red blood cell count; HGB: hemoglobin concentration. Note: multiple linear regression model adjusted for age, duration of diabetes, diabetic complications, BMI, blood pressure, HbA1c, TC, TG, ALB, SCr, smoking status, usage of hypoglycemic agents, and antihypertensive agents.

As shown in Figure 2, after adjusting for age, duration of diabetes, diabetic complications, BMI, blood pressure, HbA1c, TC, TG, ALB, SCr, smoking status, and usage of hypoglycemic agents and antihypertensive agents in the multiple linear regression analysis, ASMI positively correlated with HCT and HGB in both men and women. ASMI and RBC showed only positively correlation among men, and the trend was not significant for women (men, *r* = 0.648, *β* = 0.053, *P* < 0.001; women, *r* = 0.601, *β* = 0.024, *P* = 0.057).

### 3.4. Factors Associated with the High 10-Year CVD Risk in Patients with Diabetes Mellitus

In the univariate analysis, age, diabetic duration, BMI, smoking, drinking, SBP, TG, LDL-C, ALB, SCr, CKD, DPN, anemia, and sarcopenia were selected on the basis of biological plausibility and previous literature. Age, BMI, smoking, SBP, TG, LDL-C, SCr, CKD, and sarcopenia remained independently associated with the high 10-year CVD risk in the multivariate analysis (Table S1).

### 3.5. Association between Sarcopenia and Anemia with the High 10-Year CVD Risk in Patients with Diabetes Mellitus

**Figure 3:**
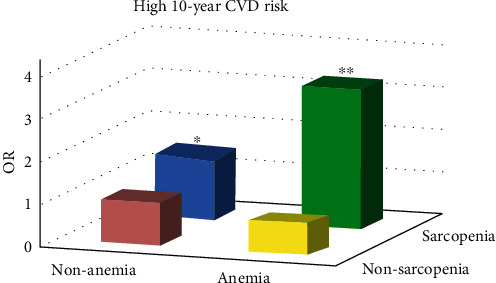
Multivariate adjusted ORs of the high 10-year CVD risk in patients with type 2 diabetes classified according to whether or not having sarcopenia or anemia. Note: logistic regression model adjusted for age, diabetes duration, diabetic complications, body mass index, HbA1c, total cholesterol, triglyceride, low-density lipoprotein cholesterol, albumin, creatinine, and smoking status. ^∗^*P* < 0.05 vs. patients in the nonsarcopenia and nonanemia group by the Cox regression analysis (OR = 1.462, 95% CI 1.085–1.972). ^∗∗^*P* < 0.001 vs. patients in the nonsarcopenia and nonanemia group by the Cox regression analysis (OR = 3.283, 95% CI 2.038–5.289).

Figure 3 illustrates multivariate adjusted ORs of the high 10-year CVD risk in patients with type 2 diabetes classified according to whether or not having sarcopenia or anemia. After adjusting for age, diabetes duration, diabetic complications, BMI, HbA1c, TC, TG, LDL-C, ALB, SCr, and smoking status, patients with sarcopenia and without anemia had a 42.9% increased risk of high 10-year CVD risk (OR = 1.462, 95% CI 1.085–1.972, *P* = 0.013), and the risk was twice as high in patients with sarcopenia and anemia than in the nonsarcopenia and nonanemia group (OR = 3.283, 95% CI 2.038–5.289, *P* < 0.001). However, there was no significant difference in the 10-year CVD risk between patients with anemia and without sarcopenia and patients in the control group (OR = 0.681, 95% CI 0.397–1.168, *P* = 0.163). When the stratified analysis was performed according to age groups, the association between anemia and sarcopenia and the high 10-year CVD risk was significant only inpatients aged 65 years and older (Table S2).

### 3.6. Baseline Characteristics of the Follow-Up Population

A total of 542 patients (282 men and 260 women) with type 2 diabetes and complete data, with an average age of 61.62 ± 10.33 years, were included in the follow-up. The median follow-up time was 24 months. Age (60.66 ± 11.93 vs. 61.62 ± 10.34 years, *P* = 0.179), BMI (24.56 ± 3.79 vs. 24.72 ± 3.28 kg/m^2^, *P* = 0.071), and sex (48.6% vs. 52.0% for men, 51.4% vs. 48.0% for women, *P* = 0.132) characteristics at baseline showed no significant difference between the 4673 patients in the cross-sectional study and the 542 patients in the follow-up study. Compared with the initial hospitalization, the FRS in the low-high group increased from 13.10 ± 2.37 to 16.56 ± 2.49 (*P* < 0.001), and the detection rate of sarcopenia increased from 16 (30.8%) to 26 (50.0%) (*P* < 0.05). Compared to the baseline, there was no significant change in the detection rate of sarcopenia in the low-low, high-low, and high-high groups at follow-up (all, *P* > 0.05). The detection rates of anemia in the four CVD risk groups increased to different degrees during the first visit after hospitalization, but there was no specific pattern in the changes in the 10-year CVD risk, and the differences were not significant.

### 3.7. Association between Changes in the 10-Year CVD Risk and Changes in ASMI and Hemoglobin

Table 2 shows the differences in the variational rates of HGB and ASMI in the follow-up between the groups with changes in the 10-year CVD risk. Compared to the low-low group, the proportion of patients with decreased ASMI was significantly high (24 (46.2%) vs. 123 (30.9%)), and the proportion of patients with increased ASMI was significantly low (12 (23.0%) vs. 152 (38.2%)) in the low-high group (*P* < 0.05). However, there was no significant difference in the proportion of patients with stabilized ASMI between the low-low and low-high groups. In patients with a low 10-year CVD risk at baseline, the risk of ASMI stabilized and ASMI decreased groups increased by more than 40% (OR = 1.470, 95% CI (0.579–3.749)) and more than 2 times (OR = 3.263, 95% CI (1.315–8.100)) than that in the ASMI increased group, respectively. Except for the high-low group, there was no significant difference in the trends of ASMI between the other three groups. No significant difference was found in the proportion of HGB changes between the low-low and low-high groups or the high-high and high-low groups.

## 4. Discussion

Our study examined the effects of sarcopenia and anemia on the 10-year risk of CVD in patients with type 2 diabetes in Fujian Province, China. Sarcopenia was associated with an increased risk of anemia in both men and women with type 2 diabetes, and the presence of both substances had an additive effect on the 10-year CVD risk. The proportion of patients with ASMI decreased in the low-high group increased significantly during follow-up; however, the decrease in ASMI was a predictor of an increased risk of 10-year CVD.

This study found that sarcopenia was associated with anemia in patients with type 2 diabetes. To the best of our knowledge, most studies on sarcopenia focus on its association with infection, fall, and weakness [28, 29], and very few studies have examined the association between sarcopenia and anemia, specifically in patients with type 2 diabetes. Previous studies have shown that both sarcopenia and anemia are both associated with malnutrition and are significant manifestations of malnutrition [14, 30]. Although the exact mechanism by which sarcopenia is associated with anemia is still uncertain, several reasons may account for this association. Loss of appetite, poor chewing, and inadequate intake of nutrients, particularly protein, is common in people with sarcopenia [31, 32], which can lead to anemia. Multiple studies have shown that an elevated serum level of IL-6 was positively correlated with various markers of weakness, such as slow walking speed, low muscle strength, and anemia [33, 34]. Hawkins et al. found that loss of muscle mass can lead to chronic inflammation and ultimately chronic anemia [35]. Moreover, diabetes increases oxidative stress and is involved in senility-induced sarcopenia. Meanwhile, oxidative stress also accelerates the loss of red blood cells in various systemic diseases, which is a potential mechanism of pathology-related anemia [36]. In addition, gender-based differences in the association between ASMI and RBC levels were observed in this study, which may be related to known gender-based differences such as the increase in RBC due to increased muscle mass in men or the effect of hormone replacement therapy on postmenopausal women with type 2 diabetes [37].

This study confirmed that sarcopenia was independently associated with an increased risk of CVD in diabetic patients and that decreased muscle mass could independently predict an increased risk of CVD in 10 years. Sarcopenia has been proven to be associated with CVD risk factors such as impaired vascular endothelial function, arteriosclerosis, hypertension, metabolic syndrome, and chronic heart failure in the general population and is also associated with an increased risk of CVD [38–41]. Despite the high HDL and low DBP and TG levels in the sarcopenia group in this study, BMI was not adjusted for any of the above indicators, failing to reflect the true level of patients with sarcopenia. Our findings are consistent with the findings of several previous studies [42, 43]. The difference is that previous studies usually looked at the actual incidence of CVD, while this study used FRS to assess the risk of CVD. The advantage is that a number of patients have been followed. Currently, the mechanisms of sarcopenia and CVD have not been clarified, but patients suffering from muscle loss often have a large amount of visceral fat or abnormal fat distribution [44]. The Framingham Heart Study showed that excess visceral fat is a predictor of CVD through the secretion of fat cell factors and the impact of other vascular active substances on cardiac metabolism [45]. In addition, Lim et al. proposed that sarcopenia be involved in the occurrence and development of CVD through mitochondrial dysfunction, oxidative stress, insulin resistance, inflammatory factors, and other mechanisms [46]. Bellanti et al. identified a marker of the increased oxidative stress cycle in sarcopenia that was associated with the risk of CVD in sarcopenic obesity [9]. When we performed a stratified analysis by age group, the association between anemia and sarcopenia with the high 10-year CVD risk was significant only in elderly patients (age ≥ 65). However, in the younger group (age < 65), an underestimation of the association may have occurred as a result of the relatively lower prevalence of sarcopenia.

No independent association was found between anemia and the high 10-year CVD risk. Previous studies on the association of anemia with CVD have been controversial. Some of the findings suggested that anemia was associated with an increased CVD risk in patients with diabetic nephropathy, renal anemia, hypertension, or congestive heart failure [13, 47, 48]. However, the National Health and Nutrition Examination Survey (NHANES) II study showed no significant association between anemia and cardiovascular mortality [49]. Our study is the first to demonstrate that the coexistence of sarcopenia and anemia has an additive effect on the 10-year CVD risk in diabetic patients. Argilés et al. concluded that anemia reduces the quality of life and life expectancy in individuals with advanced cancer, especially in patients with sarcopenia, suggesting a potential cumulative effect of sarcopenia and anemia on adverse outcomes [50]. However, given the limitations of the retrospective studies, this conclusion could not be verified in the follow-up.

There were several limitations to this study. First, the cross-sectional study prevented us from inferring a causal relationship between sarcopenia and anemia. Second, an uncontrolled selection bias could have been present due to the recruitment of participants from inpatients. Furthermore, data on grip strength and gait speed were not available in this study, which would have strengthened the analysis. In future studies, muscle function should be considered in the determination of sarcopenia. The specific type of anemia could not be determined without analysis of reticulocytes, ferritin, vitamin B12, and folic acid; bone marrow examination; and other tests. In addition, objective cardiovascular data such as the carotid artery, cardiac color ultrasound, or cardiovascular events could not be provided. Due to the short follow-up period, a 10-year risk assessment based on a Framingham score for CVD was selected instead of a prognostic assessment. Thus, the risk of CVD may not be fully reflected. Despite these limitations, our research has potential value. The strengths of this study are as follows: (1) This study had a large sample size, with a cross-sectional design and follow-up conducted successively; adjustments were made for various potential confounders, increasing the credibility of the results; (2) DEXA was used for body composition measurements to provide objective evidence.

In conclusion, the current study provides additional evidence to support the association between sarcopenia, anemia, and the 10-year CVD risk in patients with type 2 diabetes. Hence, there is a need for a range of public health interventions focused on muscle loss and anemia in patients with diabetes.

## Figures and Tables

**Table 1:** Baseline characteristics according to the presence of sarcopenia and gender.

	Men (*N* = 2271)			Women (*N* = 2402)		
	Nonsarcopenia (*N* = 1200)	Sarcopenia (*N* = 1071)	*P*	Nonsarcopenia (*N* = 1766)	Sarcopenia (*N* = 636)	*P*
Age (year)	56.68 ± 12.30	62.36 ± 12.60	<0.001	61.58 ± 10.79	62.63 ± 11.44	0.039
Diabetic duration (year)	6.92 ± 6.13	7.79 ± 6.81	0.001	8.99 ± 7.01	8.60 ± 7.30	0.240
BMI (㎏/㎡)	26.17 ± 3.03	22.23 ± 2.60	<0.001	25.92 ± 3.74	21.56 ± 3.09	<0.001
Smoking (%)	435 (45.9%)	377 (44.2%)	0.459	40 (2.9%)	3 (6.0%)	0.006
Drinking (%)	183 (19.4%)	150 (17.5%)	0.315	17 (1.2%)	1 (2.0%)	0.056
HT (%)	597 (49.8%)	504 (47.1%)	0.201	1024 (58.0%)	312 (49.1%)	<0.001
SBP (mmHg)	137.14 ± 20.65	134.3 ± 19.2	0.001	139.07 ± 21.59	137.82 ± 21.06	0.208
DBP (mmHg)	81.73 ± 11.41	77.98 ± 10.48	<0.001	77.79 ± 11.00	76.19 ± 10.66	0.002
HbA1c (%)	9.38 ± 2.51	9.58 ± 2.77	0.072	8.91 ± 2.30	9.25 ± 2.53	0.004
TC (mmol/L)	4.67 ± 1.34	4.42 ± 1.32	<0.001	4.82 ± 1.26	4.86 ± 1.26	0.516
TG (mmol/L)	2.07 ± 2.02	1.52 ± 1.31	<0.001	1.96 ± 1.59	1.59 ± 1.12	<0.001
LDL-C (mmol/L)	2.83 ± 0.98	2.75 ± 1.03	0.055	2.93 ± 1.06	3.01 ± 1.10	0.104
HDL-C (mmol/L)	1.07 ± 0.32	1.1 ± 0.36	0.011	1.18 ± 0.36	1.26 ± 0.38	<0.001
ALB (g/L)	39.94 ± 5.56	38.14 ± 5.05	<0.001	39.41 ± 4.5	38.7 ± 4.44	0.001
FPG (mmol/L)	8.92 ± 3.80	8.67 ± 3.92	0.133	8.54 ± 3.58	8.68 ± 4.00	0.434
SCr (umol/L)	78.59 ± 44.36	77.98 ± 54.17	0.772	63.54 ± 49.68	58.00 ± 43.62	0.014
UACR < 30 (mg/g)	748 (69.1%)	587 (63.6%)	0.028	950 (62.2%)	308 (55.7%)	0.018
UACR ≥ 30 (mg/g)	335 (30.9%)	336 (36.4%)		578 (37.8%)	245 (44.3%)	
eGFR (mL/min/1.73 m^2^)	99.64 ± 24.00	94.07 ± 26.28	0.182	87.33 ± 30.27	92.33 ± 25.86	0.384
CKD, *N* (%)	224 (18.7%)	217 (20.3%)	0.338	369 (20.9%)	115 (18.1%)	0.129
DR, *N* (%)	209 (17.4%)	181 (16.9%)	0.745	379 (21.5%)	122 (19.2%)	0.225
DPN, *N* (%)	462 (38.5%)	483 (45.1%)	0.001	813 (46.0%)	277 (43.6%)	0.281
OADs (%)	897 (76.1%)	803 (76.8%)	0.699	1430 (82.9%)	498 (80.1%)	0.107
Insulin (%)	408 (34.2%)	356 (33.6%)	0.760	689 (39.5%)	231 (36.8%)	0.236
*β*-Blocker (%)	67 (7.0%)	63 (7.3%)	0.764	116 (8.4%)	42 (8.9%)	0.703
ACEI/ARB (%)	228 (23.8%)	185 (21.6%)	0.273	361 (26.0%)	86 (18.3%)	0.001
CCB (%)	258 (26.9%)	193 (22.5%)	0.031	443 (31.9%)	113 (24.0%)	0.001
Statin (%)	87 (9.0%)	72 (8.4%)	0.606	153 (10.9%)	40 (8.4%)	0.122
MCH (pg)	30.68 ± 2.24	30.56 ± 2.43	0.204	29.89 ± 2.38	29.91 ± 2.69	0.837
MCHC (g/L)	344.86 ± 14.45	342.59 ± 15.35	<0.001	339.7 ± 14.64	340.28 ± 15.53	0.399
MCV (fL)	89.38 ± 5.63	89.54 ± 6.06	0.518	88.33 ± 5.76	88.16 ± 6.43	0.569
RDW (%)	13.53 ± 1.22	13.78 ± 1.37	0.055	13.62 ± 1.35	13.72 ± 1.44	0.140
HCT (L/L)	0.41 ± 0.05	0.39 ± 0.06	0.002	0.37 ± 0.04	0.36 ± 0.05	0.008
RBC (10^12^/L)	4.59 ± 0.61	4.35 ± 0.65	0.026	4.19 ± 0.5	4.13 ± 0.55	0.025
HDW (g/L)	26.51 ± 3.2	26.60 ± 3.36	0.502	26.43 ± 3.45	26.42 ± 3.57	0.987
HGB (g/L)	139.39 ± 17.99	131.81 ± 19.81	<0.001	124.47 ± 15.58	122.15 ± 16.89	0.003
Anemia (%)	138 (11.5%)	258 (24.1%)	<0.001	245 (13.9%)	125 (19.7%)	0.001
FRS	12.02 ± 4.44	12.41 ± 4.20	0.076	14.69 ± 5.16	14.54 ± 5.18	0.619
CVD high risk (%)	263 (31.2%)	248 (34.5%)	0.167	29 (2.6%)	11 (3.1%)	0.620

Note: values are number (percentage) or mean standard deviation. Abbreviations: BMI: body mass index; HT: hypertension; SBP: systolic blood pressure; DBP: diastolic pressure; TC: total cholesterol; TG: triglycerides; LDL-C: low-density lipoprotein cholesterol; HDL-C: high-density lipoprotein cholesterol; ALB: serum albumin; FPG: fasting plasma glucose; SCr: serum creatinine; UACR: urinary albumin creatinine ratio; eGFR: estimated glomerular filtration rate; CKD: chronic kidney disease; DR: diabetic retinopathy; DPN: diabetic peripheral neuropathy; OADs: oral antidiabetic drugs; ACEI: angiotensin-converting enzyme inhibitor; ARB: angiotensin receptor blocker; CCB: calcium channel blocker; MCH: mean corpuscular hemoglobin; MCHC: mean corpuscular hemoglobin concentration; MCV: mean corpuscular volume; RDW: red cell volume distribution width; HCT: hematocrit; RBC: red blood cell count; HDW: hemoglobin distribution width; HGB: hemoglobin concentration; FRS: Framingham risk score; CVD: cardiovascular disease.

**Table 2:** Association between changes in the 10-year CVD risk and changes in ASMI and HGB in patients with diabetes.

	Low-low *N* = 399	Low-high *N* = 52	*P*	OR(95% CI)^a^	High-low *N* = 27	High-high *N* = 64	*P*	OR (95% CI)
HGB decreased	108 (27.1%)	13 (25.0%)	0.809	1.217 (0.490,3.025)	11 (40.7%)	20 (31.3%)	0.329	——
HGB stabilized	228 (57.1%)	29 (55.8%)		1.217 (0.449,3.900)	16 (59.3%)	40 (62.5%)		——
HGB increased	63 (15.8%)	10 (19.2%)		1	——	4 (6.3%)		——
ASMI decreased	123 (30.9%)	24 (46.2%)	0.046	3.263 (1.315,8.100)^b^	10 (37.0%)	27 (42.2%)	0.876	1.630 (0.294,9.031)
ASMI stabilized	123 (30.9%)	16 (30.8%)		1.470 (0.579,3.749)	7 (25.9%)	14 (21.9%)		0.518 (0.113,2.373)
ASMI increased	152 (38.2%)	12 (23.0%)		1	10 (37.0%)	23 (35.9%)		1

Abbreviations: CVD: cardiovascular disease; HGB: hemoglobin concentration; ASMI: appendicular skeletal muscle mass index; OR: odds ratio; CI: confidence interval. ^a^Logistic regression model adjusted for age, diabetes duration, diabetic complications, body mass index, HbA1c, total cholesterol, triglyceride, low-density lipoprotein cholesterol, albumin, creatinine, and smoking status. ^b^*P* < 0.05.

## Data Availability

The data sets generated during and/or analysed during the current study are not publicly available but are available from the corresponding author on reasonable request.

## Supplementary Materials

Supplementary Materials Table S1: variables associated with high 10-year CVD risk in patients with diabetes. Table S2: stratified analysis of the association between sarcopenia and anemia with the high 10-year CVD risk according to age groups. Table S3: baseline characteristics of patients with and without follow-up.
