# Supplementary material for: Additive Effect of Sarcopenia and Anemia on the 10-Year Risk of Cardiovascular Disease in Patients with Type 2 Diabetes
Source: J Diabetes Res. 2022 Jan 24;2022:2202511. doi: 10.1155/2022/2202511 (PMC8803444; doi:10.1155/2022/2202511)
Supplement: Supplementary Materials — Table S1: variables associated with high 10-year CVD risk in patients with diabetes. Table S2: stratified analysis of the association between sarcopenia and anemia with the high 10-year CVD risk according to age groups. Table S3: baseline characteristics of patients with and without follow-up. [file 2202511.f1.zip › Table S1.docx]

| **Table S1 Variables associated with high 10-year CVD risk in patients with diabetes** | | | | |
| --- | --- | --- | --- | --- |
| Variables | High 10-year CVD risk | | | |
|  | Univariate analysis | Multivariate analysis | | |
|  | P | OR | 95%CI | P |
| Age | ＜0.001 | 1.167 | 1.146-1.188 | ＜0.001 |
| Diabetic duration | ＜0.001 | 0.993 | 0.973-1.013 | 0.480 |
| BMI | ＜0.001 | 1.102 | 1.057-1.148 | ＜0.001 |
| Smoking | ＜0.001 | 10.489 | 8.125-13.541 | ＜0.001 |
| Drinking | ＜0.001 | 1.194 | 0.803-1.778 | 0.381 |
| SBP | ＜0.001 | 1.037 | 1.030-1.044 | ＜0.001 |
| HbA1c | 0.449 | —— | —— | —— |
| TC | 0.895 | —— | —— | —— |
| TG | ＜0.001 | 1.711 | 1.500-1.951 | ＜0.001 |
| LDL-C | ＜0.001 | 3.143 | 2.282-4.328 | ＜0.001 |
| ALB | ＜0.001 | 1.022 | 0.991-1.053 | 0.163 |
| SCr | ＜0.001 | 1.005 | 1.003-1.008 | ＜0.001 |
| CKD | ＜0.001 | 1.685 | 1.215-2.338 | 0.002 |
| DR | 0.342 | —— | —— | —— |
| DPN | ＜0.001 | 1.235 | 0.960-1.590 | 0.101 |
| Anemia | ＜0.001 | 1.231 | 0.853-1.777 | 0.267 |
| Sarcopenia | ＜0.001 | 2.149 | 1.595-2.897 | ＜0.001 |

Abbreviations: CVD, cardiovascular disease; OR, odds ratio; CI, confifidence interval; BMI, body mass index; SBP, systolic blood pressure; TC, total cholesterol; TG, triglycerides; LDL-C, low-density lipoprotein cholesterol; ALB, serum albumin; SCr, serum creatinine; CKD, chronic kidney disease; DR, diabetic retinopathy; DPN, diabetic peripheral neuropathy.
