# Supplementary material for: Additive Effect of Sarcopenia and Anemia on the 10-Year Risk of Cardiovascular Disease in Patients with Type 2 Diabetes
Source: J Diabetes Res. 2022 Jan 24;2022:2202511. doi: 10.1155/2022/2202511 (PMC8803444; doi:10.1155/2022/2202511)
Supplement: Supplementary Materials — Table S1: variables associated with high 10-year CVD risk in patients with diabetes. Table S2: stratified analysis of the association between sarcopenia and anemia with the high 10-year CVD risk according to age groups. Table S3: baseline characteristics of patients with and without follow-up. [file 2202511.f1.zip › Table S2.docx]

| **Table S2 Stratified analysis of the association between sarcopenia and anemia with the high 10-year CVD risk according to age groups** | | | | |
| --- | --- | --- | --- | --- |
|  | Age < 65 | | Age ≥ 65 | |
|  | OR (95%CI)^a^ | P | OR (95%CI) | P |
| Control | 1 (ref) | 0.352^b^ | 1 (ref) | ＜0.001^b^ |
| Anemia | 0.381 (0.129, 1.131) | 0.082 | 0.743 (0.377, 1.460) | 0.392 |
| Sarcopenia | 0.824 (0.492, 1.382) | 0.464 | 2.135 (1.424, 3.201) | ＜0.001 |
| Sarcopenia with anemia | 0.821 (0.296, 2.278) | 0.705 | 4.769 (2.578, 6.823) | ＜0.001 |

Abbreviations: CVD, cardiovascular disease; OR, odds ratio; CI, confifidence interval.

^a^Logistic regression model adjusted for age, diabetes duration, diabetic complications, BMI, HbA1c, TC, TG, LDL-C, ALB, SCr, smoking status.

^b^ P-value for test of trend of odds.
