# Supplementary material for: Additive Effect of Sarcopenia and Anemia on the 10-Year Risk of Cardiovascular Disease in Patients with Type 2 Diabetes
Source: J Diabetes Res. 2022 Jan 24;2022:2202511. doi: 10.1155/2022/2202511 (PMC8803444; doi:10.1155/2022/2202511)
Supplement: Supplementary Materials — Table S1: variables associated with high 10-year CVD risk in patients with diabetes. Table S2: stratified analysis of the association between sarcopenia and anemia with the high 10-year CVD risk according to age groups. Table S3: baseline characteristics of patients with and without follow-up. [file 2202511.f1.zip › Table S3.docx]

| **Table S3 Baseline characteristics of patients with and without follow-up** | | | |
| --- | --- | --- | --- |
|  | Non follow-up | Follow-up | P |
|  | N=4131 | N=542 |  |
| Age（year） | 60.49±12.09 | 61.62±10.34 | 0.223 |
| Male (%) | 1992(48.2%) | 279(51.4%) | 0.036 |
| Diabetic duration（year） | 7.09±6.75 | 9.85±7.46 | ＜0.001 |
| BMI（㎏/㎡） | 24.52±3.83 | 24.72±3.28 | 0.391 |
| Smoking（%） | 782(24.0%) | 73(17.7%) | 0.004 |
| Drinking（%） | 331(10.2%) | 20(4.9%) | 0.001 |
| HT（%） | 2125(51.5%) | 312(57.7%) | 0.007 |
| SBP（mmHg） | 137.17±20.63 | 138.36±22.18 | 0.212 |
| DBP（mmHg） | 78.71±11.13 | 77.97±10.93 | 0.140 |
| HbA1c（%） | 9.26±2.52 | 9.01±2.42 | 0.029 |
| TC（mmol/L） | 4.69±1.32 | 4.79±1.2 | 0.094 |
| TG（mmol/L） | 1.84±1.62 | 1.85±1.61 | 0.891 |
| LDL-C（mmol/L） | 2.87±1.05 | 2.93±1.00 | 0.208 |
| HDL-C（mmol/L） | 1.14±0.36 | 1.17±0.35 | 0.078 |
| ALB（g/L） | 39.10±4.98 | 39.61±4.73 | 0.026 |
| FPG（mmol/L） | 8.71±3.81 | 8.51±3.54 | 0.248 |
| SCr（umol/L） | 70.00±49.48 | 69.51±48.52 | 0.829 |
| UACR＜30（mg/g) | 2266(62.7%) | 327(69.1%) | 0.006 |
| UACR≥30 (mg/g) | 1348(37.3%) | 146(30.9%) |  |
| eGFR (ml/min/1.73㎡) | 103.85±36.21 | 103.18±36.06 | 0.701 |
| CKD, N (%) | 818(19.8%) | 107(19.7%) | 0.974 |
| DR, N (%) | 788(19.1%) | 103(19.0%) | 0.968 |
| DPN, N (%) | 1773(42.9%) | 262(48.3%) | 0.017 |
| OADs（%） | 3161(78.3%) | 467(87.8%) | ＜0.001 |
| Insulin（%） | 1450(35.5%) | 234(43.5%) | ＜0.001 |
| β-blocker（%） | 243(7.4%) | 45(10.9%) | 0.013 |
| ACEI/ARB（%） | 724(22.2%) | 136(33.2%) | ＜0.001 |
| CCB（%） | 876(26.8%) | 131(31.8%) | 0.033 |
| Statin（%） | 311(9.5%) | 41(9.8%) | 0.818 |
| HGB (g/L) | 129.77±18.6 | 128.86±18.77 | 0.281 |
| Anemia (%) | 687(16.6%) | 79(14.6%) | 0.224 |
| Sarcopenia (%) | 1531(37.1%) | 176(32.5%) | 0.004 |
| FRS | 13.27±4.95 | 14.42±4.46 | 0.103 |
| CVD high-risk（%） | 498(18.3%) | 53(16.1%) | 0.311 |

Note: Values are number (percentage) or mean standard deviation.

Abbreviations: BMI, body mass index; HT, hypertension; SBP, systolic blood pressure; DBP, diastolic pressure; TC, total cholesterol; TG, triglycerides; LDL-C, low-density lipoprotein cholesterol; HDL-C, high-density lipoprotein cholesterol; ALB, serum albumin; FPG, fasting plasma glucose; SCr, serum creatinine; UACR, urinary albumin creatinine ratio; eGFR, estimated glomerular filtration rate; CKD, chronic kidney disease; DR, diabetic retinopathy; DPN, diabetic peripheral neuropathy; OADs, oral antidiabetic drugs; ACEI, angiotensin converting enzyme inhibitor; ARB, angiotensin receptor blocker; CCB, calcium channel blocker; HGB, hemoglobin concentration; FRS, Framingham risk score; CVD, cardiovascular disease.
